# Some Criticisms of Medical Fees

**Published:** 1915-05-08

**Authors:** 


### The Hospital

The Workers' Newspaper of Administrative Medicine and Institutional Life, Administration, National Insurance and Health.

No. 1507, Vol. LVIII. SATURDAY, MAY 8, 1915. PRICE ONE PENNY

### SOME CRITICISMS OF MEDICAL FEES.

The prominence into which the war has forced some of the activities of the medical profession has brought with it a not unnatural curiosity in reference to the fees paid to doctors for services of a more or less obviously public nature; and the satisfaction of curiosity has led to some measure of criticism. The most recent experience of this order is related to the announcement of the sums received by certain physicians and surgeons who have been retained by the Admiralty as expert advisers in the departments of medicine and surgery. The sum of £5,000 a year, it is suggested, is excessive, and this especially seeing that it leaves the fortunate beneficiaries free to continue to earn fees in private practice. Our own view is that neither of these contentions is well founded, and that the money spent in this fashion is wisely spent and in the public interest. On the question of private practice we now have an official statement to the effect that while this is not absolutely forbidden it is not allowed in any degree to interfere with an officer's duties; in other words, whatever opportunity for other work there may be, the Navy has the first claim, and its claim may be an exhaustive one. Those who hint the contrary ought to present proofs that private ends have been served at the expense of the public interest. Nothing short of this will influence anyone who knows the standard of honour which governs the conduct of honest men, and for our *confrères* we may surely put in this essentially modest claim.

No doubt it is true that £5,000 a year is a sum largely in excess of the annual income of the majority of even successful medical practitioners. Yet, without pretending to any personal knowledge on the question, we feel pretty certain that it is less than the earnings of at least some of the physicians and surgeons now engaged in the Admiralty service. We have little doubt, in other words, that some of these gentlemen are actually losers by the appointments, and that in a financial sense they would have been better off had they continued their ordinary professional activities. Then it must be remembered that for a consulting physician or surgeon to leave home and to decline private consultations for a period of many months, or possibly years, is a very serious decision. It makes a break which is not readily repaired, and at the end of the interval he may return to find that former associations are not always easily renewed. There is a risk, in short, that even when peace restores the conditions of civil practice, the effect of absence will continue in the shape of a diminished income. We wish not to overstate the case, but we are sure that in some instances the consideration we have just proposed has considerable influence. Again, it may be fairly recalled that the gentlemen in question stand in the most prominent rank in the profession, and that to have reached this position means the spending of many years in work which, though essential as a means of preparation for large responsibilities, carries but scanty remuneration. Further, we are sure it will be the wish of the British people that our sailors and soldiers shall have in the hour of their need the best possible medical and surgical assistance, and that anything less than this will not satisfy the national conscience. No medical practitioner would desire to utilise this wish to his own personal profit, but it is right that those who are summoned to the duty shall be reasonably compensated for the sacrifice of their private interests.

We hold no brief for any individual and we do not pretend to say that every choice made by the authorities would be endorsed by the voice of the profession, but we are sure that with few, if any, exceptions the gentlemen in question have little to gain financially from their appointments, and that those who are best acquainted with all the circumstances are satisfied that the fees paid to them are wholly reasonable.

So far, if we seem to have rested the argument largely on personal grounds, our excuse must be that there has been, if not personal attack, at least personal criticism, and some of it not quite in the open day. But the appointments and emoluments here under consideration can be defended on other and broader grounds. There are several reasons why the national service wishes the best advice and help, and one of these is that in the end it is the least expensive. To pay what is necessary to obtain it is therefore a true economy. As a general principle such a doctrine is beyond dispute, and there would be no difficulty in illustrating its value by the history of our Army now fighting on the Continent. The testimony of soldiers who have no particular disposition to view the activities of doctors with a favourable eye and who have had experience of other wars is unanimous in favour of the proposition that for the health and fighting value of the troops they are largely debtors to the Medical Services. Not only lives but money has been saved by medical efficiency.

If it is remarked that it is not the doctors who receive the big fees who are in the fighting-line the ready reply is that neither is the Commander-in-Chief. Every organised service needs its thinking units as well as its executive hands. Let each have due honour, but let it not be forgotten that the central, directing, and inspiring force is an essential condition of efficiency at all points, and that if this fails everything fails. The gifts which create it are not common, and in the very nature of things, therefore, they have their price. This price in the present instance can, we are convinced, be defended even on grounds which are purely utilitarian and economic.

